# Refraction-corrected backscatter tensor imaging of excised porcine ventricular myocardium

**DOI:** 10.1121/10.0014034

**Published:** 2022-09-12

**Authors:** John M. Cormack, Marc A. Simon, Kang Kim

**Affiliations:** 1 Center for Ultrasound Molecular Imaging and Therapeutics, Department of Medicine, University of Pittsburgh Medical Center, Pittsburgh, Pennsylvania 15261, USA; 2 Division of Cardiology, Department of Medicine, University of California San Francisco, San Francisco, California 94117, USA jmc345@pitt.edu, marc.simon@ucsf.edu, kangkim@pitt.edu

## Abstract

Backscatter tensor imaging (BTI) is performed on excised porcine right- and left-ventricular myocardium to estimate the transmural myofiber orientation. Calculation of the backscatter spatial coherence employs measured sound speeds of the myocardium and the fluid that separates the tissue from the imaging array to account for effects of refraction during the delay-and-sum beamforming calculation. Compared to the assumption of a homogeneous sound speed in the imaging region, accounting for refraction yields significantly increased average spatial coherence as well as contrast of spatial coherence between the along- and across-fiber directions, thus improving sensitivity of BTI for myofiber orientation estimation.

## 1. Introduction

Backscatter tensor imaging (BTI) is an ultrasound imaging technique that estimates the orientation of microstructure in fibrous tissues by detecting variation with direction of the spatial coherence of received echoes.^1^ The spatial coherence of echoes from the tissue is proportional to the alignment of the linear array with the fiber direction at the ultrasound focal spot.^2^ Coherent plane wave compounding^3^ (PWC) is employed in BTI to synthesize a focus throughout the imaging region, thereby reducing the number of transmits required compared to using focused waves to obtain the images.

Benchtop and *in vivo* experimental configurations often comprise two or more horizontal layers. In these configurations, the ultrasound pulses in transmit and receive are subjected to distinct propagation speeds as well as refraction through the interfaces between layers. Refraction-correction for ultrasound imaging of layered regions has been applied to soft tissue^4,5^ and bone.^6^

In this letter, we report BTI of excised right- and left-ventricular porcine myocardium using measured sound speeds to account for refraction of the transmitted and reflected pulses through the fluid-tissue interface. Refraction-correction significantly increases both the average spatial coherence and the anisotropy of the spatial coherence, i.e., contrast between the along- and across-fiber directions, which indicate superior beamforming and a larger effect of the microstructure on the backscattered echoes, respectively. Thus, refraction-correction significantly improves the sensitivity of BTI compared to the assumption of a homogeneous imaging region.

## 2. Methods

### 2.1 Imaging configurations

#### 2.1.1 Reference

**Fig. 1.**
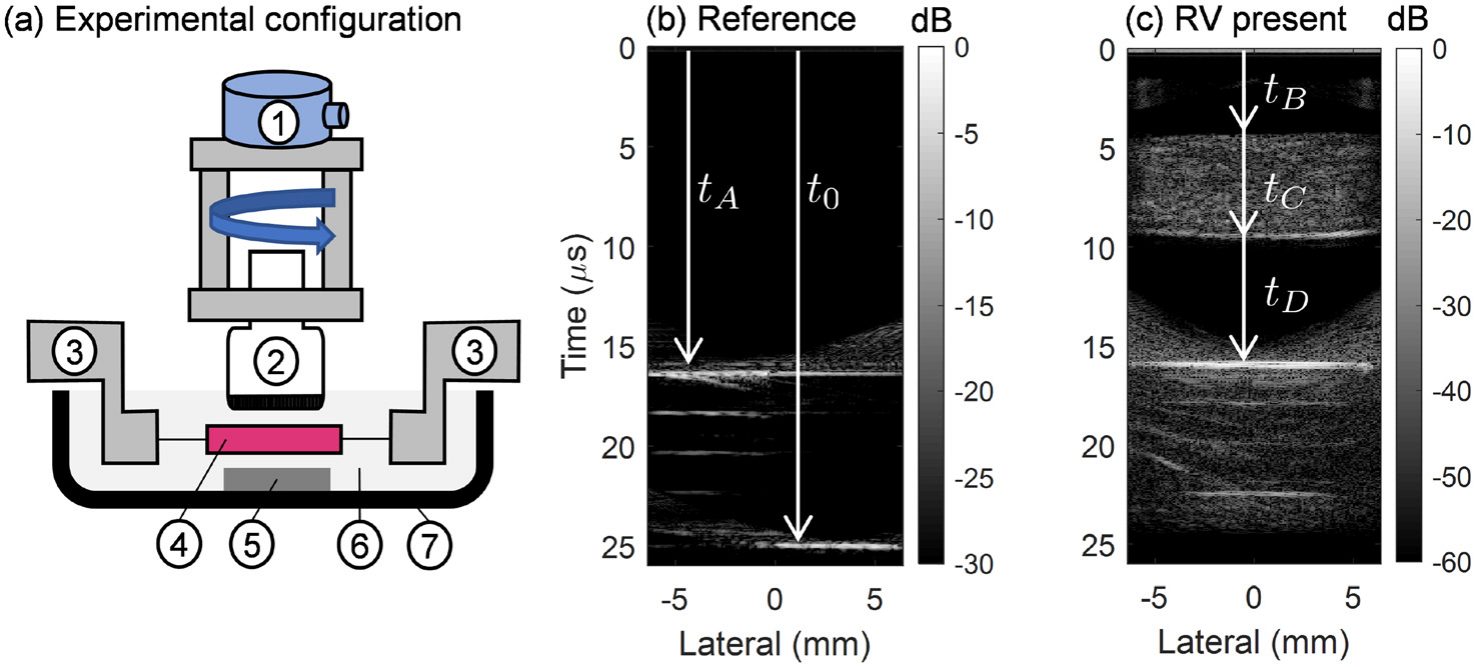
(a) Schematic of the experimental configuration: (1) motorized rotation stage, (2) ultrasound linear array, (3) biaxial tester actuators, (4) myocardium sample, (5) aluminum reference reflector, (6) PBS bath, and (7) POM fluid tank. (b) Ultrasound image of the reference configuration; (c) image with the right-ventricular (RV) myocardium sample present. Delay-and-sum beamforming of these images employed a constant sound speed of 1540 m/s, but the depth axis is given in terms of time. Arrival times used to calculate *c*_1_ and *c*_2_ using Eqs. (5) and (6) are indicated in (b) and (c).

An image is first obtained using a single un-steered plane wave of two reference reflectors with a measured separation distance of 6.34 mm, submerged in phosphate buffered saline (PBS), using a commercial linear array transducer (L22-14v, Verasonics, Kirkland, WA) controlled by a programmable ultrasound platform (Vantage, Verasonics). The transmitted waveform consists of a four-cycle pulse with center frequency of 18 MHz, sampled at 62.5 MHz. The reference reflector near to the imaging array is the upper surface of a piece of flat aluminum that rests on the bottom of the tank that contains the fluid. The far reference reflector is the bottom of the tank, which is composed of POM (Delrin). A representative B-mode image of the reference imaging configuration is shown in Fig. 1(b). The time-of-flight of echoes received from each reference reflector are used to calculate the speed of sound in the PBS as described in Sec. 2.3.

#### 2.1.2 Backscatter tensor imaging

BTI scans are performed on one sample each of excised right- and left-ventricular porcine myocardium. Samples were cut from the corresponding ventricular free wall with approximate dimensions of 20 × 20 mm and thinned to thickness *h* < 5 mm. With care taken to ensure that the position of the aluminum reference reflector is maintained, the myocardium is positioned between the imaging array and the aluminum reference reflector [Figs. 1(a) and 1(c)]. The sample is suspended using a small amount of tension with a biaxial testing machine (BioTester, Cellscale, Waterloo, Canada) to ensure that the surface of the sample is approximately parallel with the linear array as is necessary for BTI and application of Eqs. (1) and (3) below. The BTI acquisitions are modeled after the protocol of Papadacci *et al.*:^1^ a rotational scan of the linear array about the imaging (*z*) axis is performed by a motorized rotation stage (Velmex, Rochester, NY) that is controlled by the programmable ultrasound platform. Images are obtained in steps of 5° over 360°. Each image in the rotational scan employs coherent plane wave compounding (PWC)^3^ with 41 plane wave transmissions and steering angles ranging from −20° to 20° in steps of 1°.^1^ PWC is employed instead of traditional focused transmissions to reduce the number of transmits needed for transmural BTI.^1^ In addition to radio frequency (RF) signals corresponding to echoes from the myocardium, in each image, echoes from the near reference reflector are obtained. Arrival times of echoes from the tissue and the near reference reflector in this configuration are used to compute the sound speed in the myocardium as described in Sec. 2.3. Beamformed RF data from the myocardium is used to estimate the transmural fiber orientation as described in Sec. 2.4.

### 2.2 PWC time delays in a layered medium

**Fig. 2.**
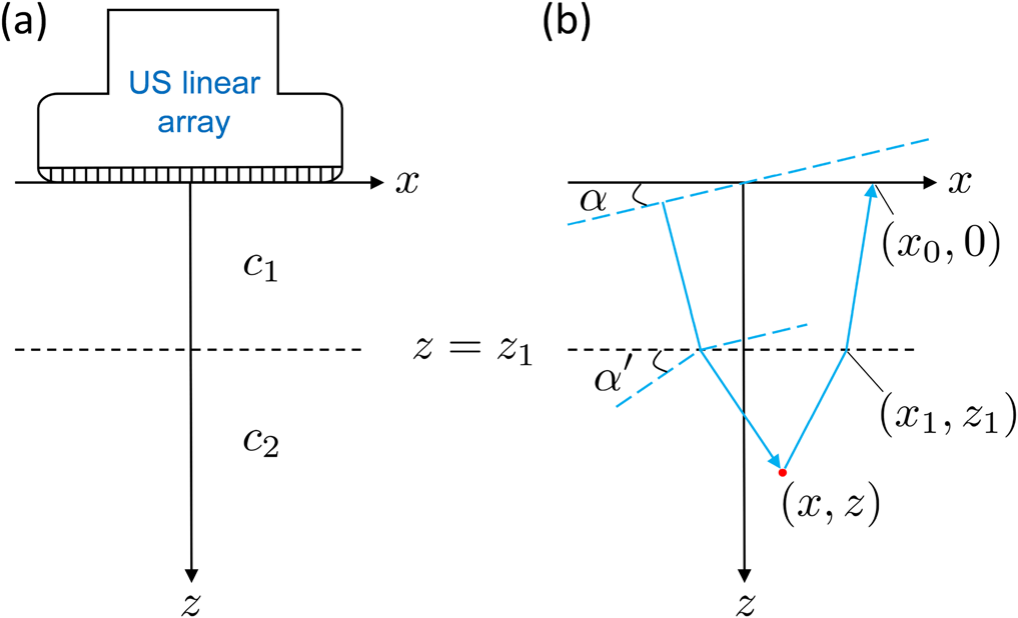
Schematic of the two-layered imaging region. (a) An ultrasound linear array is located along the *x* axis, and the two layers with differing sound speeds are separated by an interface at *z* = *z*_1_. (b) Illustration of the propagation path of a transmitted plane wave with propagation direction defined by the angle *α* and the reflected ray path back to the imaging array. Blue dashed lines are lines of constant phase, and blue solid arrows indicate propagation direction.

The linear imaging array of ultrasound transducers is located along the *x* axis and excites waves traveling in the positive *z* direction (Fig. 2). The interface between the PBS and myocardium is represented by a horizontal layer at *z* = *z*_1_. The location of a scatterer within the myocardium is given by (*x*, *z*). It is assumed that the transmitted pulse propagates through the imaging region as a planar wave and that the echoes return to the array as spherically spreading wavefronts.

A plane wave with steering angle *α* relative to the *x* axis is transmitted by the linear array. The time-of-flight of the transmitted wave to the scatterer at $\left(x,z>z_{1}\right)$ is

$$\tau_{\text{TX}}=\frac{1}{c_{1}}\left(x\;\text{sin}\;\alpha +z_{1}\;\text{cos}\;\alpha \right)+(z-z_{1})\sqrt{\frac{1}{c_{2}^{2}}-\frac{\;{\text{sin}}^{2}\alpha }{c_{1}^{2}}}\;(z>z_{1}).$$ (1)

The first term in Eq. (1) corresponds to travel time from the array to the interface and is identical to Eq. (4) in Ref. 3. The second term in Eq. (1) accounts for change in propagation direction (refraction) and sound speed in the second layer. Note that the expression under the square root in Eq. (1) can also be written as $\;{\text{cos}}^{2}(\alpha \prime )/c_{2}^{2}$, where the plane wave propagates at an angle α′ relative to the *x* axis after refraction through the interface [Fig. 2(b)].

The receive delays are found from the time-of-flight of an acoustic ray emanating from the scatterer location at (*x*, *z*) and intersecting with the ultrasound linear array at $\left(x_{0},z=0\right)$. The lateral intersection point *x*_1_ of such a ray with the interface at *z*_1_ is found from numerical solution of a quartic equation,^7^

$$\frac{{\left(x_{1}-x_{0}\right)}^{2}}{c_{1}^{2}\left[z_{1}^{2}+{\left(x_{0}-x_{1}\right)}^{2}\right]}-\frac{{\left(x_{1}-x\right)}^{2}}{c_{2}^{2}\left[{\left(z-z_{1}\right)}^{2}+{\left(x-x_{1}\right)}^{2}\right]}=0,$$ (2)

which follows from the law of refraction. The time-of-flight from the scatterer to the linear array is then

$$\tau_{\text{RX}}=\frac{1}{c_{1}}\sqrt{z_{1}^{2}+{\left(x_{0}-x_{1}\right)}^{2}}+\frac{1}{c_{2}}\sqrt{{\left(z-z_{1}\right)}^{2}+{\left(x-x_{1}\right)}^{2}},$$ (3)

where the two terms are the acoustic path length divided by the sound speed in each layer.

Thus, the appropriate delay to apply in a delay-and-sum coherent PWC scheme in a two-layered region of interest is

$$\tau =\tau_{\text{TX}}+\tau_{\text{RX}}.$$ (4)

Received RF data are delayed according to Eq. (4) and then compounded by summation over the set of plane wave steering angles.

### 2.3 Measurement of sound speeds

The sound speeds in the PBS and the myocardium sample are calculated using a time-of-flight based approach.^8,9^ RF data from the reference configuration images [Fig. 1(b)] are used to calculate the sound speed *c*_1_ in the PBS as

$$c_{1}=\frac{2L}{t_{0}-t_{A}},$$ (5)

where *L* is the distance between reference reflectors, measured beforehand with calipers to be *L* = 6.34 mm, and *t*_0_ and *t_A_* are the arrival times of echoes received at the linear array from the far and near reference reflectors, respectively. Echo arrival times *t*_0_ and *t_A_* from the reference reflectors are determined by finding the points in local search windows where the echo envelope amplitude reaches 50% of the pulse maximum. Arrival times *t_A_* are calculated using each of the signals received on the 31 elements with –5.5 mm <x<−2.5 mm, and times *t*_0_ are calculated using each of the signals received on the 31 elements with 2.5 mm <x<5.5 mm. Equation (5) is evaluated for each pair of *t_A_* and *t*_0_ (497 pairs), and the mean value is used in evaluation of Eq. (6) and in the BTI calculations.

After the reference measurements, the myocardium is introduced as shown in Fig. 1(c), and the sound speed *c*_2_ in the myocardium is calculated following the procedure outlined in Ref. 9. Echo times are obtained from A-lines (envelope of beamformed RF signals, not logarithmically compressed), which are calculated as functions of time for *x* = 0 in each of the 72 images of the rotational scan by assuming a constant sound speed equal to 1540 m/s. Echo times calculated in this way were found to be robust to this choice of sound speed in the range of 1500–1560 m/s. The arrival time *t_D_* from the reference reflector with the myocardium present was determined by finding the point where the echo envelope amplitude reaches 50% of the maximum. This timing criterion was found to yield the best accuracy and consistency in calculation of $t_{A}-t_{D}$ as evaluated by visual inspection of the delayed reference pulses. Echo arrival times *t_B_* and *t_C_* corresponding to the upper and lower myocardium surfaces, respectively, were defined as the point at which the A-line amplitude reached 25% of the local maximum in a search window approximately corresponding to the surface. This definition of the surface echo timing was chosen based on the resulting consistency between computed values of $t_{C}-t_{B}$, i.e., tissue thickness, and the visual identification of the tissue surface depths in B-mode images. The mean value of $t_{A}-t_{D}$ and $t_{C}-t_{B}$ over all 72 acquisitions of the rotational scan is used to compute the speed of sound in the myocardium using^8,9^

$$c_{2}=c_{1}\left(1+\frac{t_{A}-t_{D}}{t_{C}-t_{B}}\right).$$ (6)

The formulation of Ref. 9 also returns the thickness of the tissue sample, $h=(c_{2}/2)(t_{C}-t_{B})$, which was used as validation for the algorithm to determine *t_B_* and *t_C_* by comparison with the observed thickness in B-mode images.

### 2.4 Fiber orientation estimation

The interface $z_{1}=c_{1}t_{B}/2$ between PBS and myocardium is found from the time-of-flight calculation. Ultrasound RF data from the myocardium are delayed and compounded according to Eq. (4) (two-layer case), as well as the baseline case in which it is assumed that both PBS and myocardium have equal sound speeds of 1540 m/s. Spatial coherence of the backscattered echoes from a location in the myocardium is quantified by the coherence factor *C* of the beamformed data, which is computed as^1,10^

$$C=\frac{\sum_{T_{1}}^{T_{2}}{\left|\sum_{k=1}^{N}s(x_{k},t)\right|}^{2}}{N\sum_{T_{1}}^{T_{2}}\sum_{k=1}^{N}{\left|s(x_{k},t)\right|}^{2}},$$ (7)

where $s(x_{k},t)$ is the time-delayed and compounded RF signal corresponding to the *k*th element of the receive aperture (before summation over the receive aperture takes place), *N* is the number of elements in the receive aperture, and the range $T_{1}<t<T_{2}$ defines a temporal window centered at the focal time ( $T_{2}-T_{1}\approx 0.19$ *μ*s, or approximately 3.4 cycles at the RF center frequency, in this work). The coherence factor *C* is computed on the imaging axis *x* = 0 through the depth of the tissue in a moving averaging window centered at *x* = 0 and with dimensions of 1.5 × 0.25 mm in the *x* and *z* directions to compensate for heterogeneities that appear as coherent specular scatterers but are unrelated to the fiber direction in the tissue.^1^

Variations of interest in *C* with array orientation *θ* at a given depth of tissue thickness are due to the fiber direction at that depth, with maxima and minima in *C* expected to occur when the linear array is oriented parallel and perpendicular to the fiber direction, respectively.^2^ Thus, the variations of interest in *C* should have sinusoidal dependence with period of 180°, and the fiber angle at each depth is determined by a cosine fit to *C* at each *z*,

$$C\approx A_{0}(z)+A_{1}(z)\;\text{cos}\;[2(\theta -\theta_{\text{fib}})],$$ (8)

where $\theta_{\text{fib}}(z)$ is the fiber angle. Angles *θ* and $\theta_{\text{fib}}$ have units of radians in Eq. (8).

Accuracy of BTI is assessed by comparison with the ground-truth fiber angle determined from histological sectioning (Sec. 2.5). Sensitivity of BTI is evaluated for both the baseline and two-layer beamforming strategies by assessing the average coherence factor $A_{0}(z)$, the coefficient of determination *R*^2^ of the cosine fit, and the fractional anisotropy

$$\text{FA}=\frac{A_{1}}{\sqrt{(A_{0}^{2}+A_{1}^{2})/2}}.$$ (9)

A larger value of average coherence *A*_0_ indicates superior beamforming of echoes from the myocardium.^11^ Higher values of *R*^2^ and fractional anisotropy (FA) indicate a stronger effect of the microstructure on the backscattered coherence and, therefore, increased sensitivity of BTI for identification of the fiber angle through the tissue thickness.

### 2.5 Histology

Transmural fiber angle variation derived from BTI is validated against the ground truth of optical microscopy after histological sectioning. Samples were fixed in 10% neutral buffered formalin and then sectioned and stained with hematoxylin and eosin (H&E). Digital images of each section are taken with a trinocular microscope (1.08 *μ*m/pixel, field of view 2.76 mm × 2.08 mm), and the fiber angle is determined with the OrientationJ/DistributionJ toolbox^12^ in ImageJ^13^ with local window set to 40 pixels (43.2 *μ*m), minimum coherency to 2%, and minimum energy to 2%. Similar to previous studies,^14,15^ the fiber angle and dispersion are reported as the circular mean and circular standard deviation of the computed pixel-level local orientations.

## 3. Results and discussion

Sound speeds in the PBS and myocardium during the BTI scan of the RV sample were $c_{1}=1481\pm 4$ m/s and $c_{2}=1540\pm 4$ m/s, respectively. Sound speeds in the PBS and myocardium during the BTI scan of the left-ventricular (LV) sample were $c_{1}=1479\pm 3$ m/s and $c_{2}=1516\pm 5$ m/s, respectively. Sample thicknesses were 4.03±0.04 and 2.67±0.04 mm for the RV and LV samples, respectively.

**Fig. 3.**
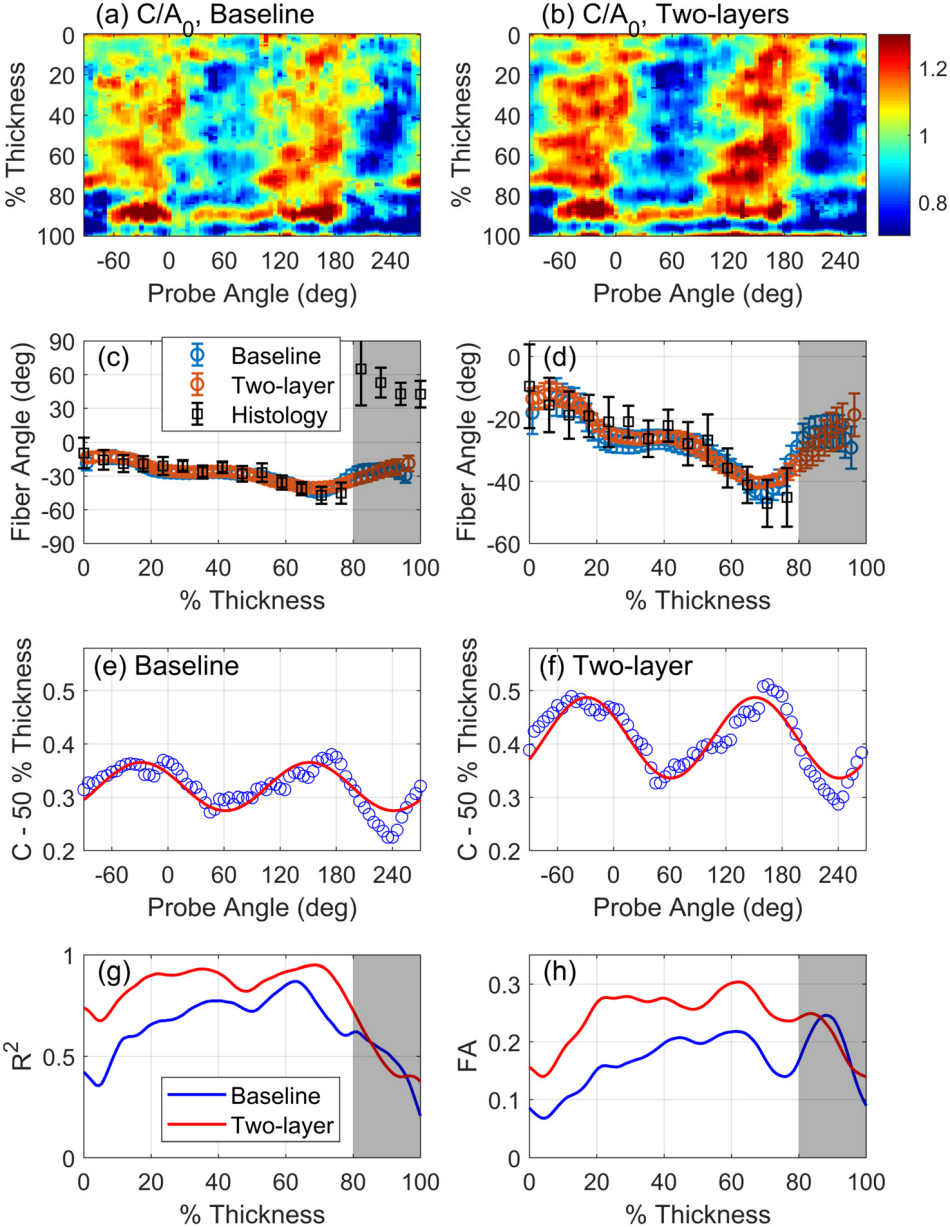
(a) and (b) Color maps of normalized coherence factor $C/A_{0}$ versus tissue thickness and probe angle for the baseline and two-layer (refraction-corrected) cases, respectively. (c) Ultrasound-derived fiber angle in both cases and ground-truth fiber angle derived from histology. (d) Expanded view of (c). (e) and (f) Examples of coherence variation with ultrasound probe angle; blue circles are measured coherence, and red curves are cosine fit according to Eq. (8). (g) Coefficient of determination *R*^2^ for the cosine fit; (h) FA computed using Eq. (9). Gray area between 80% and 100% thickness in (c), (d), (g), and (h) indicates the epicardial layer in which BTI is not effective.

Results of the BTI calculation in the baseline and two-layer (refraction-corrected) cases are presented in Table 1 for both RV and LV samples. Detailed results are presented in Fig. 3 for the RV tissue scan. While not presented or discussed in detail here, the corresponding results for the sample of LV myocardium are qualitatively identical. Normalized coherence factor $C/A_{0}$ as a function of array rotation angle *θ* and depth are presented in Figs. 3(a) and 3(b) for the baseline and two-layer cases, respectively. Depth is expressed in terms of percent thickness through the tissue, where the upper and lower surfaces of the tissue were identified as described in Sec. 2.3, and the lower surface of the tissue is the epicardium. Regions of high and low coherence correspond to the orientations *θ* of the linear array that are parallel and perpendicular to the fiber direction at that depth, respectively. Increased sharpness of the bands of high and low coherence in the two-layer case is apparent in the refraction-corrected case [Fig. 3(b)] compared to baseline [Fig. 3(a)].

**Table 1.** Summary of BTI accuracy and sensitivity metrics for the tested RV and LV tissues. †, two-sample F-test for equal variance, *p* = 0.42. ‡, two-sample F-test for equal variance, *p* = 0.28. *, paired *t*-test, *p* < 0.001. **, RV results do not include the epicardial layer from 80% to 100% thickness.

Tissue	Case	RMSE (deg)	Average *A*_0_	Average *R*^2^	Average FA
RV**	Baseline	6.2^†^	0.32*	0.68*	0.16*
	Two-layer	4.8^†^	0.43*	0.87*	0.25*
LV	Baseline	10.0^‡^	0.26*	0.51*	0.21*
	Two-layer	7.1^‡^	0.38*	0.65*	0.28*

Accuracy of the fiber angle estimation is presented in Figs. 3(c) and 3(d). Ultrasound-derived fiber orientation is in good agreement with histology for 0%–80% thickness for both baseline and two-layer cases. The region of tissue from 80% to 100%, indicated by the gray region in Figs. 3(c) and 3(d), is a region of rapidly varying and non-uniform microstructure unique to porcine RV epicardium.^16^ The rapid change in microstructure is seen in Fig. 3(c) by the abrupt jump in fiber angle of approximately 70° at 80% thickness. BTI is not effective in that region of the tissue thickness due to the rapidly changing and disorganized tissue microstructure. The LV sample exhibited smooth transmural variation in fiber orientation, with BTI able to estimate the fiber orientation throughout the thickness.

The main result of the present work is in the significant increase in sensitivity metrics for BTI throughout the tissue thickness when accounting for the slower sound speed in the PBS and for refraction through the fluid-tissue interface. Computed coherence *C* of the backscattered echoes from 50% thickness is shown versus array angle *θ* in Figs. 3(e) and 3(f) for the baseline and two-layer cases, respectively. Comparison of the cosine fit (red curves) in Figs. 3(e) and 3(f) demonstrates the increased average coherence *A*_0_ and amplitude of coherence modulation *A*_1_ in the two-layer case compared to baseline. Larger values for the average coherence *A*_0_ indicate increased accuracy of beamforming, and larger values of *A*_1_ indicate increased sensitivity of BTI to the fiber structure when the slower sound speed of the PBS and refraction are accounted for. Larger values of *A*_0_ and *A*_1_ in the two-layer case are found throughout the tissue thickness apart from the epicardial layer.

The coefficient of determination *R*^2^ for the cosine fit is shown versus tissue thickness in Fig. 3(g). Outside of the epicardial layer, the value of *R*^2^ is higher in the two-layer case (red curve) compared to baseline (blue curve), indicating that the fibrous microstructure accounts for more of the variation in coherence with probe angle when the slower sound speed in the PBS and refraction are accounted for. FA of the coherence is presented in Fig. 3(h). Higher values of FA are obtained in the two-layer case compared to baseline, indicating increased contrast of spatial coherence and increased sensitivity of BTI when the slower sound speed of the PBS and refraction are accounted for. Higher anisotropy of coherence allows for identification of the fiber orientation in tissues with less organized fiber structure, e.g., tissue with disarrayed fibers or a distribution of fiber orientations at a given location in the tissue thickness.^2^

The same metrics were computed for the sample of LV myocardium and are presented along with those for the RV sample in Table 1. Root mean square error (RMSE) values compared to histology are similar to those reported for BTI in porcine LV tissues with a two-dimensional (2D) array.^17^ Significant increases in the average transmural *A*_0_, *R*^2^, and FA are seen in both samples throughout the tissue thickness (values for the RV sample are calculated ignoring the epicardial layer from 80% to 100% thickness). Average *A*_0_ and FA are notably lower in this study compared to those of Papadacci *et al.*,^1^ who report $A_{0}\approx 0.5$ and FA≈0.4 in porcine LV myocardium. The difference is possibly due to the higher frequency used in this study (18 MHz here versus 6 MHz in Ref. 1), which is more susceptible to reduced coherence resulting from off-axis specular scattering from small heterogeneities.

This study demonstrates the benefits of accounting for the distinct sound speeds in a layered imaging region during estimation of transmural fiber orientation using BTI. For example, by using this approach, the sensitivity may be increased for BTI performed in applications for which the sample cannot contact the imaging array or be embedded in a gel, such as biaxial mechanical testing.
